# The Pectoralis Block: A Case Series of a Novel Modality for Acute Pain Control in the Emergency Department

**DOI:** 10.5811/cpcem.1408

**Published:** 2023-05-27

**Authors:** Jonathan Henry Brewer, Noah Sanders, Alexander Ayala, Arun Nagdev

**Affiliations:** * Vanderbilt University Medical Center, Department of Emergency Medicine, Division of Emergency Ultrasound, Nashville, Tennessee; † Highland Hospital, Department of Emergency Medicine, Oakland, California

**Keywords:** regional anesthesia, nerve block, pocus, procedural ultrasound, case series

## Abstract

**Introduction**: Regional anesthesia has long been used in a perioperative setting for the treatment of both pre- and postoperative pain. Recently, this skill has been brought into the emergency department (ED) as a modality for treating acute pain as the pendulum shifts away from an opioid-based armamentarium and toward a multimodal future. In this case series, we describe a way to use the pectoralis nerve block I and II in the treatment of pain with regard to breast abscesses and/or breast cellulitis managed in the ED.

**Case Series**: This paper describes three cases, all of which consist of a painful complaint in the thoracic region. The first was a patient diagnosed with a breast abscess. The second patient was diagnosed with breast cellulitis. Finally, the third patient was diagnosed with a large breast abscess that extended into the axilla. All three sustained immense relief with the pectoralis block.

**Conclusion:**

While further research is needed on a larger scale, preliminary data suggests that the ultrasound-guided pectoralis nerve block is an effective and safe modality of acute pain control in regard to breast and axillary abscesses along with breast cellulitis.

## INTRODUCTION

Breast abscesses and cellulitis are common presentations in the emergency department (ED). Adequate analgesia for abscess drainage can be difficult to obtain and is currently limited to local infiltration and/or procedural sedation.^1^–^3^ Also, post-procedural pain control with oral and intravenous (IV) medications (often involving opioids) is commonly inadequate. This can be especially concerning in breastfeeding or pregnant women, populations at higher risk for development of breast abscesses and cellulitis.^4^,^6^

The pectoralis nerve (Pecs) block I and II, originally developed for analgesia following breast surgery, may be ideal options for pain control in the patient with breast cellulitis or requiring breast abscess drainage in the ED.^5^ The Pecs I block targets the medial and lateral pectoral nerves to anesthetize the pectoralis major and minor muscles. The Pecs II block targets the upper intercostal nerves to anesthetize the skin and soft tissue overlying those muscles.^5^ In this paper, and often in the literature, both blocks together are referred to as the “Pecs block.” It is prudent to know that although there have been newer nomenclature suggestions for these blocks—namely, the “interpectoral plane block” has been suggested to replace Pecs I and the “pectoserratus plane block” has been suggested for Pecs II—we will use Pecs I and Pecs II for this case series because of the lack of current consensus regarding nomenclature.^7^

As emergency physicians become increasingly comfortable with ultrasound-guided nerve blocks, the Pecs I and II block can be an integral part of non-opioid, multimodal pain management for patients with breast cellulitis or abscesses. Herein, we present three cases of breast pain successfully managed by emergency physicians with the Pecs I and II block.

## TECHNIQUE

The Pecs I block consists of an injection into the fascial plane between the pectoralis major and minor muscles. There is debate as to whether a second injection between the pectoralis minor and serratus anterior muscles (Pecs II) is needed, as the former injection provides anesthesia to the medial and lateral pectoral nerves. The Pecs II block, on the other hand, is primarily focused in anesthetizing the upper intercostal nerves, which provides more lateral coverage. In our experience, if there is any pain or swelling to the very lateral aspect of the breast or the axilla region, it may be prudent to add the Pecs II block. However, for most of our usage, the Pecs I block provides adequate anesthesia to the breast tissue.

**Image 1:**
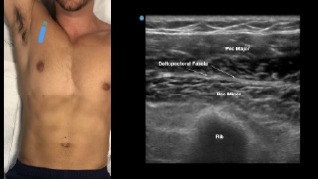
Initial probe placement for the pectoralis nerve block I and II illustrated on a model: the blue line indicates transducer, and the green dot indicates directional marker corresponding to ultrasound image.

Prior to the start of the procedure, the patient must have IV access and be placed on a cardiac monitor. After informed consent has been obtained, the patient is positioned in the supine position with the head to the contralateral side of the proposed block. The physician stands at the head of the bed above the ipsilateral breast with the ultrasound screen in direct line of sight (commonly at the level of the contralateral hip). The ultrasound probe is initially placed in the sagittal plane at the midclavicular line until the clavicle, pectoralis muscles, and axillary artery and vein are visualized. The transducer is then translated caudally until the third and fourth intercostal spaces are visualized (Image 1).

**Image 2:**
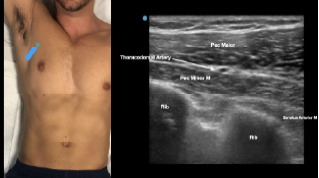
Final probe placement prior to the pectoralis nerve block I and II: blue line indicates transducer, and green dot indicates directional marker corresponding to ultrasound image.

At this point, the pectoralis major and minor muscles can be visualized. By rotating the transducer 45 degrees clockwise, the thoracoacromial artery can be identified between the pectoralis major and minor muscles. Also, the serratus anterior muscle should be identified resting just above the anechoic rib (Image 2).

**Image 3:**
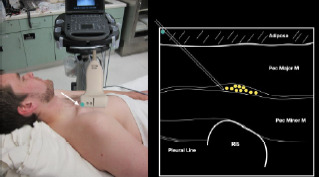
Pectoralis nerve block I injection between the pectoralis major and minor muscles: arrow on patient model indicates needle direction; the green dot indicates directional marker, and yellow dots indicate injectate within the fascial plane.

After appropriate skin disinfection, the block needle is advanced in-plane from the cephalad to caudal aspect of the patient. The needle should be advanced under clear ultrasound visualization during the entire procedure. For the Pecs I block, advance the needle to the fascial plane between the pectoralis major and minor muscles. Hydrodissection with normal saline will confirm opening of the correct fascial plane (Image 3).

*CPC-EM Capsule*

What do we already know about this clinical entity?

*The Pectoralis block has long been used for analgesia following breast surgery. It has been shown to be safe and provides effective pain control to both the skin and soft tissues of the breast*.

What makes this presentation of disease reportable?

*While regional anesthesia is established in the perioperative setting, its use in the emergency department (ED) for acute pain is fairly novel. This block provides the ED with a new approach for pain control*.

What is the major learning point?

*Due to known complications from opiates and sedation, the Pectoralis block should be considered for patients that present with painful breast complaints*.

How might this improve emergency medicine practice?

*As we attempt to shift towards a multimodal approach for analgesia, regional anesthesia can provide an effective and safe modality of pain control in the ED when compared to opiates and sedation*.

**Image 4:**
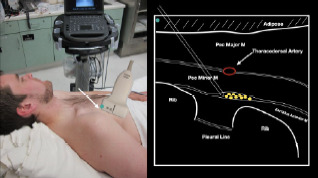
Pectoralis nerve block II injection between the pectoralis minor and serratus anterior muscles: the arrow on patient model indicates needle direction; the green dot indicates directional marker; and yellow dots indicate injectate within the fascial plane.

Anesthetic can then be gently and slowly deposited in 2–3 milliliters (mL) aliquots to a recommended amount of 15 mL per Pecs block. It is imperative to calculate your weight-based recommended dosage of anesthetic beforehand as to prevent local anesthetic systemic toxicity (LAST). If dilution is needed, the injectate can be mixed with sterile 0.9% normal saline. If the Pecs II block is going to be performed for axillary coverage, the needle is then advanced to the plane between the pectoralis minor and serratus anterior muscles (Image 4).

Of note, if both Pecs block I and II are being performed in a single injection, we recommend first depositing anesthetic in the fascial plane between the pectoralis minor muscle and serratus anterior muscle (Pecs II), since this can obscure the superficial structures.

Possible complications of the Pecs block include pneumothorax, vessel or nerve injury, and LAST. A contraindication to either block is cellulitis overlying the site of injection. Calculation of weight-based maximal anesthetic dosing should be performed prior to the block to maximize LAST prevention. For the purpose of this case series, a weight of 70 kilograms (kg) will be assumed. The duration of the block will vary based on the choice of anesthetic. In addition, all patients undergoing the Pecs block require cardiac monitoring for at least 30 minutes following injection and during the procedure. Clinicians should also be aware of ultrasound-based landmarks and the signs and symptoms of LAST. The symptoms of LAST generally appear as a progression from neurological symptoms (ie, tinnitus, metallic taste, coma, seizures) to cardiovascular symptoms (ie, ventricular dysrhythmias, conduction block, cardiovascular collapse, asystole). As a result, 20% intralipid emulsion should be readily available for any large-volume block, and all clinicians should be clearly aware of dosing protocols.

## CASE SERIES

### Case One

A 23-year-old pregnant woman presented to the ED with pain and swelling in her left breast. She had a known history of breast cancer and had been seen by her breast surgeon four days prior for mild redness. She had been placed on cephalexin without relief. A point-of-care ultrasound revealed a threecentimeter (cm) abscess on the medial aspect of her breast. The patient’s vital signs were unremarkable other than a mild tachycardia of 110 beats per minute. The surgical service came to the bedside to evaluate the patient and asked for procedural sedation for drainage. Because of ED crowding, the physician opted for a Pecs I block at the bedside with 15 mL of 0.25% ropivacaine. About 30 minutes after completion of the block, five mL of 1% lidocaine with epinephrine was used to anesthetize the skin over the abscess. The surgical team performed a bedside incision and drainage, removing 10 mL of purulent material. The patient was observed in the ED for 24 hours and then discharged with close, outpatient follow-up.

### Case Two

A 35-year-old non-pregnant woman presented to the ED with pain and redness in her left medial breast for two days. The patient was ill-appearing with the following vital signs: heart rate 120 beats per minute, respiratory rate 16 breaths per minute, blood pressure 160/85 millimeters of mercury (mm Hg) and temperature 102.6° Fahrenheit. The patient was in moderate distress and complained of 10/10 pain despite receiving 15 milligrams (mg) of IV ketorolac and four mg of IV morphine. A point-of-care ultrasound exam did not show an abscess but rather diffuse cellulitis. An ultrasound-guided Pecs I block was performed with 15 mL of 0.5% bupivacaine. The patient’s pain decreased to 2/10 at 30 minutes post block. The patient was then admitted for IV antibiotics and maintenance oral analgesia.

### Case Three

A 26-year-old non-pregnant woman presented to the ED with a six-cm axillary abscess (noted on point-of-care ultrasound). The patient was in severe distress and had vital signs notable for a heart rate of 110 beats per minute but appeared non-toxic. The patient received eight mg of morphine and 15 mg of ketorolac via IV with some relief, but she was unable to tolerate abscess drainage. A Pecs block I and II was performed under ultrasound guidance. Fifteen mL of 0.25% ropivacaine was placed between the pectoralis minor and serratus anterior muscles (Pecs II block) and 10 mL of 0.25% ropivacaine was placed between the pectoralis major and minor muscles (Pecs block I). After 30 minutes the patient’s pain was significantly decreased (3/10), allowing the physicians to place five mL of lidocaine 1% with epinephrine superficial to the abscess site. Incision and drainage was performed successfully with more than 10 mL of purulent material removed. A loop drain was left in place. The patient was discharged with antibiotics, oral pain medicine, and close, outpatient surgery follow-up.

## DISCUSSION

In our case series, the Pecs block I and II were shown to be effective methods for controlling pain from breast abscesses and/or cellulitis within the anterior breast and axilla, respectively. Single injections of 10–15 mL of local anesthetic provided excellent analgesia with varying durations based on the type of local anesthetic used. In most cases, we uaed bupivacaine or ropivacaine for longer lasting coverage. Additionally, in all relevant cases, procedural sedation was avoided, with patients tolerating additional local anesthesia as well as incision and drainage at the bedside. These cases demonstrate the utility of the Pecs block for analgesia in patients with pain from breast infections. As such, the Pecs block helps avoid procedural sedation and plays an integral role in non-opioid, multimodal analgesia in the ED.

Especially when the involvement of breast surgeons is warranted (ie, abscesses larger than five cm or adjacent to areola), offering multimodal pain control is vital while determining the ideal location and method for drainage. In settings where access to specialty surgery remains limited, such as many non-academic centers, the Pecs block can be an ideal method for pain control until the patient can be seen by a breast surgeon.

## CONCLUSION

Ultrasound-guided pectoralis nerve block I and II can provide safe and rapid analgesia for patients with painful breast abscesses and/or cellulitis. Further research is needed to determine whether both the Pecs block I and II are required to provide adequate analgesia and anesthesia for simple breast abscesses. However, these blocks can be easily performed at the bedside with a portable ultrasound machine with minimal risk to the patient. All in all, we believe that the Pecs block can serve as an integral component of multimodal, non-opioid, analgesic regimens within the emergency department.
